# Reproductive aging and elective fertility preservation

**DOI:** 10.1186/s13048-018-0438-4

**Published:** 2018-08-11

**Authors:** Rani Fritz, Sangita Jindal

**Affiliations:** 1 Department of Obstetrics, Gynecology & Women’s Health, Albert Einstein College of Medicine/Montefiore Medical Center, Minneapolis, USA; 2 Montefiore’s Institute for Reproductive Medicine and Health, New York, USA

**Keywords:** Reproductive aging- oocyte cryopreservation- reproduction

## Abstract

Reproductive aging is a natural process that occurs in all women, eventually leading to reproductive senescence and menopause. Over the past half century there has been a trend towards delayed motherhood. Postponing reproduction can increase the chance of a woman remaining involuntarily childless as well as an increase in pregnancy complications in those that do achieve pregnancy at advanced maternal age. Despite the well-documented decrease in fecundity that occurs as a woman ages, reproductive aged women frequently overestimate the age at which a significant decline in fertility occurs and overestimate the success of assisted reproductive technologies (ART) to circumvent infertility. Oocyte cryopreservation enables women to achieve genetically related offspring in the event that they desire to postpone their childbearing to an age after which a significant decline in fertility occurs or in circumstances in which their reproductive potential is compromised due to medical pathology. Available success rates and safety data following oocyte cryopreservation have been reassuring and is not considered experimental according to the American Society for Reproductive Medicine and the European Society for Human Reproduction and Embryology. This review article will focus on an evidence-based discussion relating to reproductive aging and oocyte cryopreservation.

## Background

Over the past several decades spanning from 1970 to 2014, the average age of first time mothers in the U.S. has increased 18.6% from 21.4–26.3 y/o [1, 2]. Alongside this trend, from 1970 to 2012 in the U.S. the percentage of women aged 35–44 y/o having children for the first time has increased more than five-fold from 2.5 to 13.3 per 1000 women [3]. Similar trends are also found in other parts of the world including Canada and Europe with one of the highest mean age of first time mothers seen in Italy at 30.6 y/o [4, 5]. These trends are likely related to an increase in women entering the workforce, obtaining advanced educational degrees, and greater use of contraceptive methods [6]. The declining birthrate is a concern for many developed countries. As birthrates in many industrialized countries fall below the replacement fertility rate of 2.1 live births per women, there is concern for future economic and social consequences including a decreased workforce to sustain the economy and care for the elderly population [7, 8]. It has therefore been advocated in Europe that a more liberal incorporation of assisted reproductive technologies (ART) into society can assist in achieving fertility rates towards replacement levels [8].

Women are born with a finite number of oocytes reaching their peak at 20 weeks gestation with 6–7 million and decreasing to 1–2 million at birth, 300,000–500,000 at puberty, 25,000 by 37 y/o, and approximately 1000 at menopause [9, 10]. Alongside a quantitative decline in oocyte number, there is also a qualitative decline primarily attributed to an increase in aneuploidy amongst aging oocytes. A study evaluating ploidy status of over 1300 metaphase II oocytes revealed that aneuploidy rates remain relatively stable from 20 to 34 y/o ranging between 5.2–10%, increasing to 12.5–28.1% at ages 35 y/o to 40 y/o, 50% at ages 42–43 y/o, and 100% at 45 y/o [11]. A similar trend is seen in embryo aneuploidy rates, as a study evaluating over 15,000 consecutive embryo trophectoderm biopsies with comprehensive chromosomal screening revealed a dramatic increase in embryo aneuploidy rates at ages 35–37 y/o, approaching 90% at ages 43–45 y/o [12]. The increase in oocyte and embryo aneuploidy in relation to age is likely related to a disturbance in meiotic competence due to abnormal meiotic spindle formation [13, 14].

Increase aneuploidy rates with ovarian aging results in decreased fecundity, with most epidemiological studies revealing a significant decline between 35 and 37 y/o [15]. The Hutterites are one of the most frequently referenced epidemiological cohorts. They immigrated form Switzerland and settled in the Dakotas and Montana in the 1870’s and detailed records were kept relating to their childbearing [16]. They did not practice contraception and were a relatively fertile population with an average childbirth rate of 9.6 children per woman and a low an infertility rate of 2.4%. In this cohort, a steep decline in fecundity was seen at 35-37y/o, with 1 in 10 woman stopping to reproduce by 35 y/o, 1 in 3 by 40 y/o and 7 in 8 by 45 y/o [16]. This trend revealing a significant decrease in fecundity at 35–37 y/o is consistent with multiple historical epidemiological studies [17]. Recent cohort studies reveal similar trends. A time to pregnancy cohort study evaluating the fecundability of 960 women aged 30–44 y/o trying to conceive 3 months or less revealed stable pregnancy rates of 82–88% after 12 menstrual cycles in women aged 30–35 y/o, decreasing to 71–76% between 36 and 39 y/o, and to 48–54% between 40-44y/o [18]. Interestingly, history of no prior pregnancy adversely affected fecundability rates. For example, in patients aged 38–39 y/o with a history of a prior pregnancy, 81% had a pregnancy after 12 cycles compared to 35% without a prior pregnancy, with similar trends seen in patients ≥40 y/o [18]. A major limitation of this study is that they did not report on live birth rates, as patients were only followed to a positive pregnancy test. With the known increase in miscarriage rates in older patient populations, live birth rates would have likely been significantly lower in the older patient populations. Other recent cohort studies reveal a similar trend with decreasing fecundability after 35 y/o [19, 20]. Limitations of the Hutterite study and other epidemiological studies are that they do not account for the possibility of decreasing sexual activity amongst aging women, however, a study evaluating intrauterine insemination (IUI) pregnancy rates using donor sperm amongst women with male partners suffering from azoospermia revealed a significant decrease in success rates after 35 y/o [21]. After 12 cycles, women 26–30 y/o had a success rate of 74.1%, whereas, women over 35 y/o had a success rate 53.6%. IUI success rates were defined as a positive pregnancy test and, as above, given the increase in miscarriage rates seen with advanced maternal age, if followed to live birth, the difference between both age groups would have likely widened. Another study evaluating pregnancy rates in 751 donor sperm cycles from couples with male infertility revealed a similar significant decline in pregnancy rates after 35 y/o [22].

Data from in vitro fertilization (IVF) cycles in the United States reveal a similar decrease in fecundity with advancing age. There is a persistent misconception that assisted reproductive technology can reverse the “aged biological clock” [23], however, IVF success rates decrease significantly as women enter their 5th decade of life. U.S data from the Centers for Disease Control in 2015 reveal live birth rates per cycle of IVF of 33.1% in woman under 35 years old, decreasing to 8.3% at 41–42 y/o, 3.2% at 43–44 y/o and 0.8% > 44 y/o [24]. Alongside these trends, there is an increase in the use of donor oocytes with advancing maternal age. Donor oocyte use is 10% or less ≤40 y/o, increasing to 18% at 41–42 y/o, 34% at 43–44 y/o and 71% over 44 y/o [24].

Involuntary childlessness is a major consequence of delayed motherhood. Psychologically and socially, involuntary childlessness is associated with higher rates of low self-esteem, depression, partner separation, and even higher rates of mortality [25]. There are also consequences of pregnancy at advanced maternal age to both the mother and newborn. Women ≥35 y/o are at increase risk for preeclampsia, gestational diabetes, placenta previa, placental abruption, operative deliveries including cesarean section, and even maternal deaths [26–29]. Women of advanced maternal age, particularly ≥40 y/o are also at increased risks of adverse neonatal outcomes including preterm birth, low birth weight, very low birth weight, neonatal intensive care unit admissions, fetal death, congenital anomalies, and increased risks of chromosomal abnormalities [26, 27, 29–32]. Spontaneous abortion, or miscarriage, also significantly increase with advance maternal age. Rates of clinical miscarriages are generally approximately 15%, however, increase to 20% by 35 y/o, and 50% by age 42 y/o [33]. Miscarriage rates in infertile populations following IVF are similar to those seen in the non-infertile population, as miscarriage rates following IVF are below 15% in women < 35 y/o, and rise to 30% at 40 y/o and 65% in women older than 44 y/o [24]. Although generally not life threatening, miscarriages may result in severe psychological burden. A survey evaluating patients with miscarriages revealed that 37% felt that they had lost a child, 47% felt guilt, 41% felt alone, and 28% felt ashamed [34]. This psychological burden may be more devastating in women of advanced maternal age struggling with infertility. Additionally, miscarriages following IVF in women of advanced maternal age may have adverse effects on the success of subsequent treatments as a delay of 6 months can cause a significant decrease in the quality and quantity of ovarian reserve.

Although it is well documented that advanced maternal age decreases a woman’s chance to conceive, studies from multiple countries consistently reveal that reproductive aged women have suboptimal knowledge relating to reproductive aging, particularly underestimating age-related fertility decline, and over-estimating the success of IVF [35–42]. One of the largest surveys evaluating over 10,000 women and men from 79 countries revealed an average score of 56% on a 13 item fertility knowledge questionnaire [43]. Knowledge relating to reproductive aging and IVF success outcomes have also been shown to be suboptimal amongst obstetrics and gynecology (OB/GYN) US residents. A study evaluating 238 OB/GYN residents revealed that although approximately 73% believed that a conversation relating to fertility decline with aging should be part of a well-woman exam, 47.5% underestimated the age at which a marked decline in fertility occurs, and 78.4% over-estimated the success of IVF [44].

## Premature accelerated loss of fertility, risk factors, ovarian reserve markers

Although multiple markers for ovarian reserve have been evaluated, age is likely the most predictive marker for ovarian reserve and the ability to conceive. The average age of menopause in Western countries is approximately 51 years of age [45]. Approximately 10% will experience menopause by 45 y/o, and 1% will experience primary ovarian insufficiency (POI) defined as menopause by 40 y/o [46, 47]. It is estimated that there is an accelerated decline in fertility 13 years prior to the onset of menopause, and thus approximately 10% of woman will experience an accelerated decline in fertility at around 32 y/o [46]. Ideally these patients would be easily identified so that they can be counseled on reproductive aging and the availability of elective fertility preservation, however, currently there are no optimal tests to identify these cohort of women. Obtaining an adequate medical history may identify women at risk for POI or an accelerated loss of fertility. Women diagnosed with cancer that will undergo chemotherapy or pelvic radiation, or patients that have undergone these treatments are at risk and warrant counseling with a reproductive specialist relating to elective fertility preservation. Other patients that may be at risk for accelerated fertility decline include severe endometriosis, ovarian surgery, strong smoking history, family history of early menopause, Turner Syndrome or Turner mosaic, and Fragile X pre-mutation [48–53].

Day 3 follicle stimulating hormone (FSH) alongside estradiol (E_2_), antral follicle count (AFC), and anti-mullerian hormone (AMH) are all frequently utilized markers of ovarian reserve; however they cannot predict when a significant decline in fertility will begin to occur and are more predictive of ovarian response during IVF than the ability to conceive naturally [54, 55]. Early follicular FSH and E_2_, generally drawn on day 3 of the menstrual cycle, require no advanced training to interpret and therefore can be obtained and interpreted by general practitioners; however, they have significant inter-cycle and intra-cycle variability and require a functional hypothalamic pituitary ovarian axis [56]. Day 3 elevated FSH is indicative of diminished ovarian reserve and is attributed to low E_2_ and inhibin B production from ovarian follicles thereby resulting in diminished hypothalamic suppression. Normal FSH levels in the presence of elevated E_2_ may also be indicative of low ovarian reserve as this is related to premature follicle selection and premature elevation of E_2_, thus negatively inhibiting the hypothalamus and masking diminished ovarian reserve.

AFC is the sum of all the ovarian follicles between 2 and 10 mm visualized on ultrasound. AFC count has good specificity, however, poor sensitivity for prediction of ovarian response to stimulation and pregnancy outcomes during IVF. AFC is easy to perform by a trained sonographer, however, its limitations are that it can have significant inter-observer variability and obesity can adversely affect the measurements [57].

AMH is secreted by the granulosa cells of the pre-antral and small antral follicles and likely plays a role in limiting follicular recruitment of follicles from the primordial pool, partially by attenuating their response to FSH [58]. AMH null mice reveal accelerated follicular depletion [59]. The benefits of AMH measurements are that they can be tested anytime throughout the cycle and are directly secreted form the ovary. Several studies have shown that AMH may also predict the timing of menopause [60–62]. Limitations of AMH, similar to day 3 FSH/E_2_ and AFC, are that they are more reliable to predict ovarian response and IVF outcomes rather than the ability to conceive naturally, and can be falsely elevated and decreased by many reproductive, environmental, and lifestyle factors [56, 63]. One factor common to reproductive aged women is the use of oral contraception (OCP). AMH can be falsely decreased in women using OCP’s and it is recommended to wait 3–4 months following discontinuation of OCP to measure AMH levels [56].

A recently published prospective time-to-pregnancy study evaluating the predictive value of AMH and Day 3 FSH in over 700 women aged 30–44 y/o, without a history of infertility, and attempting to conceive ≤3 months revealed that low AMH and elevated FSH were not predictive of the ability to conceive up to 12 menstrual cycles [64]. It is important to note that in the older age group (38–44 y/o), low AMH was also not predictive of time to pregnancy. A significant limitation of this study worth noting is that pregnancy was defined as a positive pregnancy test and if followed to live birth, it is possible that those in the low AMH/elevated FSH groups may have experienced pregnancy loss more often and therefore live birth rates may have differed.

## Oocyte cryopreservation

There are no known treatments to circumvent the natural decline in fecundity that occurs with ovarian aging. Using donor oocytes is an option for women unable to conceive with their own oocytes; however, this may not be acceptable to many women and is not available and/or legal in many countries. Embryo cryopreservation and OC are available options, which may enable women to become pregnant with their own oocytes after their reproductive potential has significantly diminished. The focus of the following paragraphs will focus on OC as the majority of women that delay childbearing report lack of a partner as a reason for delay [65, 66].

The first pregnancy following OC was in 1986 [67]. Over 2 decades later, in 2012, the “experimental” label for OC was removed by the American Society for Reproductive Medicine (ASRM) and the European Society for Human Reproduction and Embryology (ESHRE) [68, 69]. Factors that led to removing the “experimental” label included improved success rates of OC and reassuring safety data relating to offspring born following OC. Since the removal of the experimental label by ASRM, OC has increased 18.6% in 1 year from 6123 cycles in 2014 to 7518 cycles in 2015 [70, 71].

## Slow freeze versus Vitrification

A major advancement in OC was a shift from the slow freeze method of cryopreservation to vitrification. Vitrification is an ultra-fast cooling method for cryopreservation using high concentrations of cryoprotectants. Vitrification solidifies the oocyte to a glass-like state and minimizes ice crystal formation, more commonly seen with the slow freeze technique. The oocyte is particularly vulnerable to damage from ice crystal formation due to its high water content and its sensitive meiotic spindle apparatus [72, 73]. A randomized controlled trial reported improved oocyte survival rates, fertilization rates, and clinical pregnancy rates with vitrification compared to slow freeze [74]. Post-thaw survival rates using vitrification have been reported to be as high as 96.9% [75]. Pregnancy rates following OC have been reported to be similar to those following fresh oocyte insemination by multiple randomized controlled trials [76–78]. Although reports of high oocyte survival rates and similar pregnancy rates with OC compared to fresh insemination have been reported, these high success rates may be attributed to publication bias and therefore the success rates may not be reproducible by all centers. Data from 30,160 donor oocyte cycles reported to the Society for Assisted Reproductive Technology from 2013 to 2015 revealed that pregnancy rates from cycles following OC in donor cycles were significantly lower compared to donor cycles involving freshly inseminated oocytes [79].

## Indications for oocyte cryopreservation

Aside from preventive measures for age related fertility decline, women at risk for an accelerated rate of fertility decline due to medical reasons may benefit from OC. Women diagnosed with malignant diseases that will undergo chemotherapy and/or pelvic irradiation are at significant risk for premature ovarian failure and certainly warrant counseling on OC. Women with severe endometriosis/endometriomas may be at risk for an accelerated rate of follicular decline. A study has found that ovarian cortex from ovaries with endometriomas have significantly decreased follicular densities compared to contralateral ovaries without endometriomas [53]. In a similar study, cortex from ovaries with endometriomas had significantly more atretic early follicles compared to contralateral ovaries without endometriomas [80]. Additionally, a systematic review and meta-analysis revealed that excision of endometriomas could negatively impact ovarian reserve [81]. Women with a family history of mental retardation or a known family history of Fragile X Syndrome may benefit from Fragile X carrier testing as women with premutations (55 to 200 repeats) are at increase risk of premature ovarian failure (up to 21%) and therefore could benefit from OC [52]. Other genetic disorders include patients with Turners or Turners mosaic and those with a strong family history of premature ovarian failure. Pedigree studies have shown a familial link to idiopathic premature ovarian failure as multiple genes likely play a role that are not fully understood [82]. Women undergoing female to male gender transition often undergo bilateral salpingoophorectomy and therefore should be offered OC as pregnancies have been achieved in this cohort of patients following transition [83]. Other indications for OC include inability to produce sperm at time of ooycte retrieval, ethical dilemma to producing and cryopreserving embryos, and circumventing legal implications with embryo cryopreservation in the event of divorce.

## Age at which to freeze oocytes

A question frequently asked by both practitioners and patients is the ideal age at which to freeze oocytes. Although no good answer exists as every patient has unique biological and social circumstances, there is some evidence available to guide the discussion. Similar to IVF, success rates following OC may be related to the age at which the oocytes were frozen. A retrospective multicenter study spanning an 8-year time period evaluating 1468 women undergoing elective oocyte cryopreservation for reasons other than a diagnosis of cancer revealed that 191 (13%) presented to thaw and inseminate their oocytes [84]. Of those that did, the oocyte survival rate in women ≤35 y/o and ≥ 36 y/o were 94.6% vs. 82.4%, respectively [84]. Additionally, differences in live birth rates for women undergoing OC ≤ 35 y/o and ≥ 36 y/o were significantly different, with 50% and 22.9%, respectively [84]. In this study, age was associated with success even in the cohort of women undergoing OC ≤ 35 y/o, with live birth rates of 100% ≤ 29 y/o, 45% at 30–34 y/o, 28.5% at 35–39 y/o, and 3.7% at ≥40 y/o [84]. Despite a clear trend in success rates with age at which a woman undergoes OC, the majority of women that undergo elective OC are doing so after a significant decline in fertility begins to occur, with three studies revealing average ages between 36 y/o and 38 y/o [65, 84, 85].

In women considering OC, a frequent question is how many oocytes should be frozen. Although there is no precise answer to this question as every women presenting for OC will be at a different age and have different future childbearing desires, there is some data to guide a discussion. Clinical pregnancy rate per oocyte thaw have been shown to range between 4.5 and 12% [68]. A recent mathematical model based on live birth rates from a single institution from women with presumably normal ovarian reserve undergoing intracytoplasmic sperm injection (ICSI) due to male factor infertility or tubal factor infertility, revealed that for women 34, 37, and 42 y/o, they would need to freeze 10, 20, and 61 mature oocytes respectively to have a 75% likelihood of having at least 1 live birth [86]. If incorporating this or another model into a OC counseling session, it is important to discuss the possibility of needing multiple controlled ovarian hyperstimulation (COH) cycles to obtain the number of oocytes the patient desires. For example, based on the above model, a 37 y/o may feel comfortable with freezing 20 mature oocytes for a 75% chance of at least 1 live birth, however, obtaining 20 mature oocytes may require 2 or 3 COH cycles. Additionally, it must be stressed to patients undergoing OC that although mathematical models exist to guide a discussion of how many oocytes to freeze in order to assure a certain percentage of success, OC is not an 100% insurance policy to achieve a genetically related offspring and that there is a possibility of failure despite “adequate” amounts of frozen oocytes.

## Cost considerations of OC

Cost of oocyte cryopreservation may vary widely from infertility center to infertility center and is an important part of a counseling session for patients desiring elective OC as this is frequently not covered by insurance for reasons other than a medical indication. Although OC incurs an expense, if performed early it may be more cost-effective and successful for women desiring to postpone their childbearing to their late 30’s or early 40’s. One model has shown that OC would be more cost-effective and successful if performed at 35 y/o in women looking to prolong their childbearing until 40 y/o, whereas another model revealed that OC was most cost-effective and successful in achieving a live birth if performed by 37 y/o [87, 88]. In these models, the assumption is that the patient will return for 1 child at an older age, however, many patients may desire > 1 child. It is important to discuss with patients how many children they desire, as the more children they desire, the more cost-effective it may be to undergo OC at a younger age. Other important cost considerations that must be discussed with patients are the annual cost of storing the oocytes, cost of thawing and inseminating oocytes via ICSI, and eventual uterine transfer of the embryos. Additionally, ethical considerations of OC must be extensively discussed, documented, and consented including duration of storage of oocytes and disposition of oocytes in the event of death or incapacitation of the patient.

## Risks of OC

Risks of OC must be discussed with patients undergoing the procedure. Major complications for patients are low (< 1%) and include risks during oocyte retrieval including infection, damage to organs, blood loss, ovarian torsion, and risks related to anaesthesia [89–91]. A more common risk that must be discussed is ovarian hyperstimulation syndrome (OHSS), a complication specific to COH. Although mild and moderate OHSS is burdensome for the patient, severe OHSS is potentially life-threatening and most concerning. The risk varies depending on the definition and study, but has generally been reported to be < 5% [92]. Severe OHSS is more common following COH with embryo transfer resulting in a pregnancy and is rare in the absence of pregnancy. Women undergoing OC, by definition, will not undergo a fresh embryo transfer and therefore the risk of severe OHSS is significantly less than patients undergoing IVF/ICSI with subsequent fresh embryo transfer. A study evaluating 4052 oocyte donors revealed a risk of moderate to severe OHSS of < 1%, which was eliminated with the use of a GnRH antagonist protocol and GnRH agonist trigger [93].

A frequent concern for women undergoing OC is whether there is any risk to offspring born following OC. Although data are limited and more long-term data are needed, there does not appear to be any risk to offspring related to OC. A study of 1027 children born from 804 pregnancies using vitrified oocytes compared to 1224 children from 996 pregnancies with IVF using fresh oocytes revealed no significant differences in obstetrical outcomes, gestational age at delivery, birthweight, birth defects, APGAR scores, or perinatal mortality between cryopreserved and fresh oocytes [94]. Another study evaluating 936 infants born from both vitrified and slow freeze oocytes revealed an anomaly rate of 1.3%, similar to the anomaly rate in the general population [95]. Additionally rates of embryo aneuoploidy have been reported to be similar following oocyte cryopreservation and IVF using fresh oocytes [96, 97].

### Education and increasing awareness towards reproductive aging and elective OC

It is well documented that reproductive aged woman frequently underestimate the impact that increasing age has on the ability to conceive and overestimate the success of IVF to circumvent infertility. Sup-optimal physician counseling may be partly related to this as a study evaluating US OB/GYN residents reveals that they too have suboptimal knowledge relating to reproductive aging and IVF success rates [44]. A study by Hodes-Wertz et al. evaluating 183 women who underwent elective oocyte cryopreservation revealed that only 25% heard of the procedure from their OB/GYN, and 79% wished that they had undergone the procedure at an earlier age, with the most common reason for delay being unaware of the procedure or feeling that the technology was not readily available [65].

Although elective OC is generally not covered by private health insurances in the U.S. or government health policies in Europe, attitudes and policies relating to covering elective OC are shifting. Large companies such as Facebook and Apple are offering their employees elective oocyte cryopreservation [98]. Additionally, some countries such as Israel endorse elective OC for prevention of age related fertility decline and consider it “preventative medicine” [99].

Improved methods to increase awareness relating to reproductive aging and the availability of OC are warranted. A greater emphasis by OB/GYN’s to counsel patients on age related fertility decline during annual exams and implementing these discussions as part of sexual education may help increase awareness. Additionally using media as a platform to discuss these important issues may be of importance as 42.3% of those that underwent elective OC in the above study by Hodes-Wertz et al. heard about the technology through media outlets [65]. Having a separate clinic to counsel women on reproductive aging may also be of benefit. A Fertility Assessment and Counseling Clinic was established in Copenhagen, Denmark, with a goal of providing pro-fertility individual assessment and guidance to women and men [100]. At the time of publication > 1200 women and men attended the clinic and filled out a survey. Ninety-nine percent of women found the clinic helpful with approximately 70% wanting to know how long they could postpone their childbearing, and after consultation, 35% of women stating that they would advance the age at which to become pregnant.

## Conclusion

The adverse effect of reproductive aging on fecundity has been well documented for centuries. With delayed motherhood becoming a norm in many countries, involuntary childlessness will likely continue to rise. This may not only be detrimental to a woman’s overall well-being but may also have adverse economic consequences to future generations. Despite this, reproductive aged women are frequently unaware and underestimate the effects of reproductive aging, and/or overestimate the success rates of IVF to circumvent infertility, particularly in older aged women. Increase awareness and education at earlier ages are warranted so that women can make informed decisions relating to their reproductive potential. For the first time in history, OC provides a way of increasing a woman’s reproductive autonomy for those desiring genetically related children towards the end of their reproductive lifespan. The past several decades has seen an increase in effective available methods to delay childbearing including greater access to contraception and safe pregnancy terminations, however, an integral part of a woman’s reproductive autonomy is pro-fertility knowledge. Discussions relating to reproductive aging and oocyte cryopreservation will enable woman to make informed decisions relating to their reproductive potential and increase their reproductive autonomy.

## Abbreviations

AFC: Antral follicle count
AMH: Anti-mullerian hormone
COH: Controlled ovarian hyperstimulation
E_2_: Estradiol
FSH: Follicle stimulating hormone
ICSI: Intracytoplasmic sperm injection
IUI: Intrauterine Insemination
IVF: In vitro fertilization
OB/GYN: Obstetrics and Gynecology
OCP: Oral contraception
OHSS: Ovarian hyperstimulation syndrome
